# The Influence of Effective Energy on Computed Tomography Number Depends on Tissue Characteristics in Monoenergetic Cardiac Imaging

**DOI:** 10.1155/2012/150980

**Published:** 2012-10-09

**Authors:** Satoshi Okayama, Tsunenari Soeda, Yasuhiro Takami, Rika Kawakami, Satoshi Somekawa, Shiro Uemura, Yoshihiko Saito

**Affiliations:** First Department of Internal Medicine, Nara Medical University, 840 Shijo-cho, Kashihara, Nara 634-8522, Japan

## Abstract

*Purpose*. To evaluate the influence of effective energy on computed tomography (CT) number in monoenergetic images (MEIs). *Methods*. Three bottle phantoms filled with water, oil, and a contrast agent were scanned at 100 and 140 kVp tube energy with a dual-source CT scanner. Cardiac dual-energy CT data was collected from 17 patients. CT numbers were measured in the 3 phantom materials and in the left ventricular cavity, myocardium, pericardial fat, and vertebral bone in MEIs from 40 to 190 keV. *Results*. In the phantoms, the mean CT number increased in oil whereas it decreased in the contrast agent as the energy level increased (*P* < 0.001). In clinical subjects, the mean CT numbers for the left ventricular cavity, myocardium, and vertebral bone were highest in the 40 keV images (*P* < 0.001) and decreased as the energy level increased. In contrast, the CT number for pericardial fat was lowest in the 40 keV images (*P* < 0.001) and increased with increasing energy. *Conclusions*. The influence of effective energy on CT number varies with material and tissue type in monoenergetic cardiac imaging, which could evaluate tissue characteristics through assessment of the changes in CT number associated with effective energies.

## 1. Introduction

Dual-source computed tomography (CT) consists of 2 X-ray tubes with 2 corresponding detectors arranged at an angular offset, which produces CT images with high temporal resolution [1]. Dual-source CT can simultaneously operate each tube at a different tube potential, thus generating dual-energy images at a temporal resolution of 140 ms [2].

Dual-energy computed tomography (DE-CT) can generate composite images and monoenergetic images by calculating the 2 image datasets acquired using polyenergetic X-rays at high and low tube energy. In monoenergetic imaging, optimal contrast-enhanced images can be produced with less beam-hardening artifacts. As previously reported, image quality in monoenergetic imaging is heavily influenced by the energy level, and optimization of the effective energy for individual patients is thus important for appropriate utilization of monoenergetic coronary CT angiography [3]. However, there are few reports available on cardiac monoenergetic imaging. The purpose of this study was to evaluate the influence of effective energy on CT number in cardiac monoenergetic images.

## 2. Materials and Methods

### 2.1. Phantom Study

#### 2.1.1. Phantoms

Three 350 mL plastic bottles (diameter, 6 cm) were filled, 1 with water, 1 with salad oil, and 1 with a contrast agent. The contrast agent, Iopamidol (370 mg I/mL; Iopamiron 370, Bayer Healthcare Pharmaceuticals, Berlin, Germany), was diluted with water, such that the solution reached the CT number similar to that of the ascending aorta (approximately 500 HU) in routine coronary CT angiography. The bottle phantoms were hermetically sealed and placed on the CT bed parallel to the *z*-axis of the CT system.

#### 2.1.2. CT Data Acquisition

All CT images were acquired using a dual-source CT scanner (Somatom Definition Flash; Siemens Healthcare, Erlangen, Germany). Each detector provides for data acquisition with 64 detector rows of 0.6 mm collimation. Together with a *z*-flying focal spot, this allows simultaneous acquisition of data in 2 × 128 slices. For cardiac dual-energy CT, 1 tube was operated at 153 mAs/rot at 140 kVp with Selective Photon Shield (Sn 140 kVp), and the other was operated at 180 mAs/rot at 100 kVp. The gantry rotation time of the system is 280 ms, and a temporal resolution of 140 ms can be achieved in the center of gantry rotation. No new iterative reconstruction was used; instead, CT images were reconstructed using conventional filtered back projection. Polyenergetic and monoenergetic images were used with a medium-smooth kernel (B25f) and a dedicated dual-energy convolution kernel (D30f), respectively. Both 100 and Sn 140 kVp image sets were reconstructed at a slice thickness of 0.75 mm and at 0.4 mm slice increments. These technical conditions were consistent with those of routine cardiac dual-energy CT.

#### 2.1.3. CT Data Reconstruction

The DE-CT data were reconstructed using a workstation (Syngo, Siemens Healthcare). We generated composite 120 kVp images by merging 30% and 70% of the 100 and Sn 140 kVp spectra, respectively. Many modern CT scanners have been operated at a tube energy of approximately 120 kVp (effective energy, 60–90 keV) for routine standard imaging, because this setting yields good image quality [4]. Therefore, composite 120 kVp images were used as the basic images for coronary CT angiography. For this study, polyenergetic images were defined as 100 and Sn 140 kVp images and composite 120 kVp images. Monoenergetic images were then generated at 40, 70, 100, 130, 160, and 190 keV.

#### 2.1.4. Analysis of Phantom Data

All of the polyenergetic and monoenergetic images were reconstructed into 5 sections consisting of 2.0 mm thick transaxial images. Two cardiologists (S. Okayama and T. Soeda) analyzed the images using open-source software (OsiriX, version 4.0) and reached consensus. In polyenergetic images, the relationships between tube energy and CT number were evaluated and in monoenergetic images between effective energy and CT number. All images were visually evaluated at a window width and level of 600 HU and 100 HU, respectively. Circular regions of interest (ROIs) of 15 cm^2^ were placed at the center of the bottle phantoms in the 5 sections, respectively, and their locations were accurately correlated among all images. Mean CT numbers were measured.

### 2.2. Clinical Study

#### 2.2.1. Subjects and Methods

**Figure 1:**
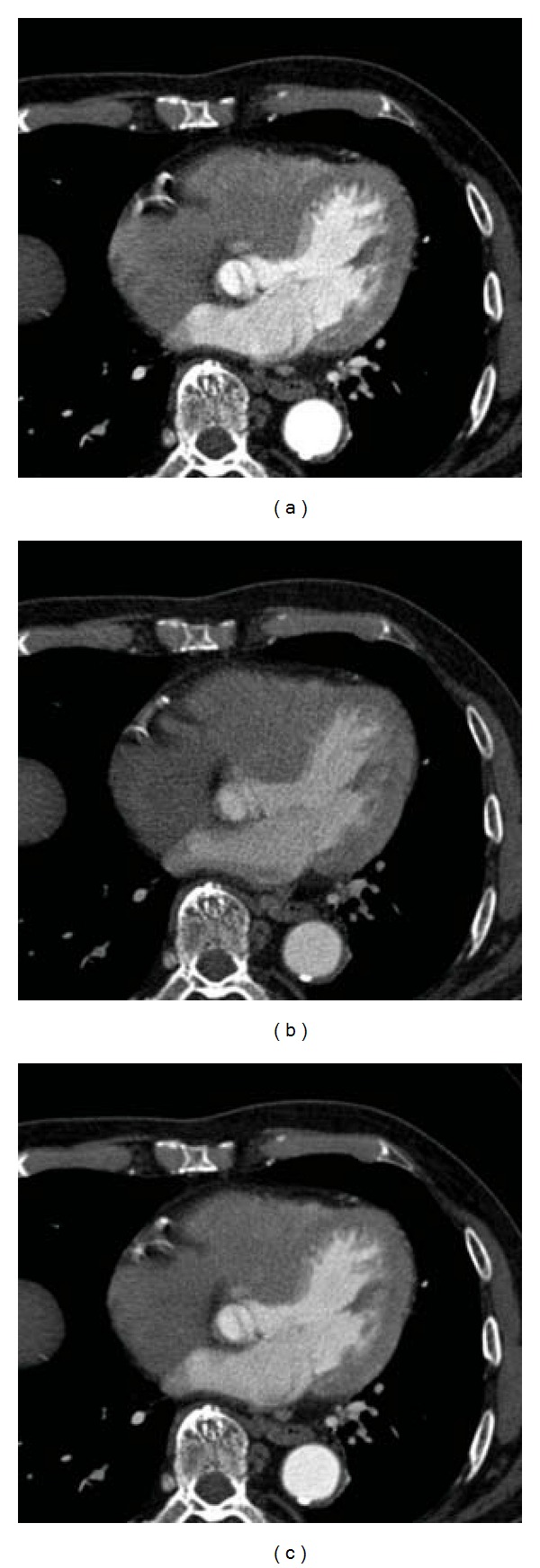
A representative case excluded from the analysis. (a) 100 kVp image. (b) Sn 140 kVp image. (c) Composite 120 kVp. Motion artifacts and misalignment between the 100 and Sn 140 kVp images are observed.

Data from 20 consecutive patients that met inclusion criteria who underwent cardiac dual-energy CT between April and November 2011 at Nara Medical University were retrospectively evaluated. Inclusion criteria required that patients did not have coronary artery stenoses considered severe enough for revascularization therapy and no prior percutaneous coronary intervention or coronary artery bypass grafting. Among the 20 patients, 3 patients with a heart rate that increased to more than 70 bpm during CT scanning were excluded from the analysis due to motion artifacts and misalignment between the 100 and Sn 140 kVp images (Figure 1). Consequently, we analyzed a total of 17 patents. Cardiac dual-energy CT was routinely performed at our institution, in nonobese patients with a regular heart rate below 70 bpm; others underwent single-energy CT. The present study proceeded according to the principles set forth by the Declaration of Helsinki [5].

**Figure 2:**
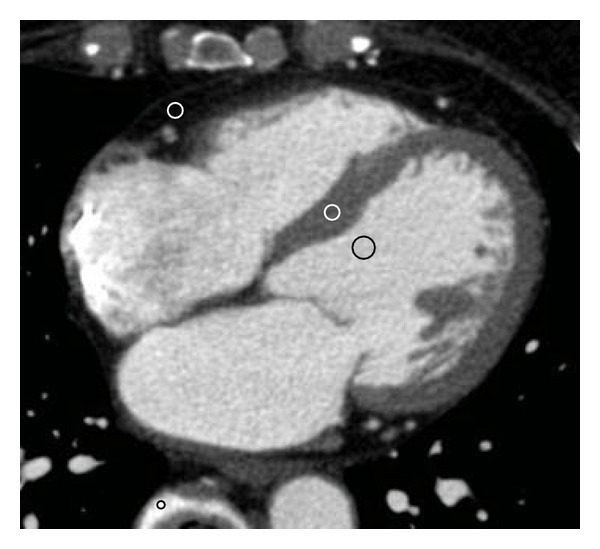
Setting regions of interests (ROIs). Circular ROIs were placed on axial images at the left ventricular cavity (area, 0.5 cm^2^), interventricular septal myocardium and pericardial fat (areas, 0.1–0.2 cm^2^), and vertebral compact bone (area, 0.05–0.1 cm^2^).

Methods of CT data acquisition and reconstruction were as described for the phantom study. To clarify how the CT numbers change according to the energy levels in the left ventricular cavity, myocardium, pericardial fat, and vertebrae, the same 2 cardiologists analyzed all images using OsiriX. All images were visually evaluated at a window width and level of 600 HU and 200 HU, respectively. Circular ROIs were placed on axial images at the left ventricular cavity (area, 0.5 cm^2^), interventricular septal myocardium and pericardial fat (areas, 0.1–0.2 cm^2^), and vertebral compact bone (area, 0.05–0.1 cm^2^), as shown in Figure 2. The left ventricular papillary muscles and intramyocardial arteries were carefully avoided during ROI placement in both the cavity and septum. The locations of ROIs were correlated among all images in each patient, and mean CT numbers were measured.

#### 2.2.2. Statistical Analysis

All data were analyzed using Prism 5.0 (GraphPad Software Inc., La Jolla, CA, USA) and are presented as mean ± SD. Relationships between energy levels and CT numbers were evaluated using repeated-measures ANOVA with the Tukey-Kramer post-hoc test. A *P*-value of <0.05 was considered statistically significant.

## 3. Results

### 3.1. Phantom Study

**Figure 3:**
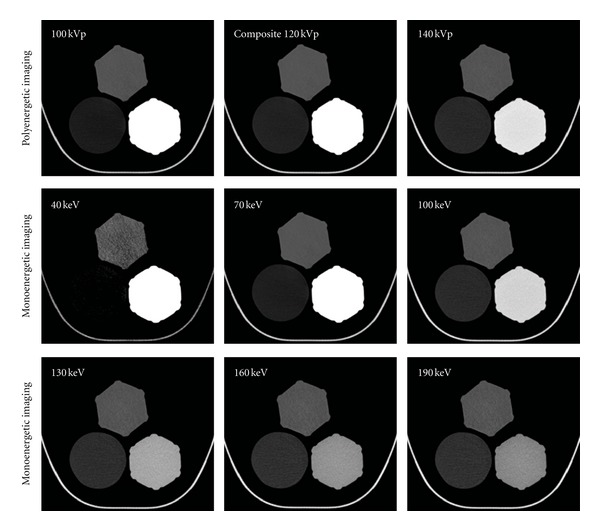
Phantom images; each panel illustrates (counterclockwise from the top) phantoms filled with water, oil, and the contrast agent. All images are displayed with a window width of 600 HU and window level of 100 HU. Top row: typical polyenergetic images. The left panel is a representative 100 kVp image; oil appears slightly darker, and the contrast agent slightly brighter compared to the composite 120 kVp (middle panel) and Sn 140 kVp images (right panel). Water is similar among the 3 images. Bottom rows: typical monoenergetic images with increasing effective energies are shown left to right. As effective energy increased, oil became brighter, while the contrast agent darkened. Water remained unchanged throughout all effective energy levels.

The mean CT numbers of the 3 phantom materials were compared among the 100, Sn 140, and composite 120 kVp polyenergetic images. Typical polyenergetic images of the phantoms are shown in Figure 3 (top row). Oil appeared slightly darker and the contrast agent slightly brighter in the 100 kVp images, compared to both the composite 120 kVp and the Sn 140 kVp images. Water appeared similar among all the 3 images. The CT numbers for the 3 materials according to tube energy for the polyenergetic images are shown in Table 1. We confirmed that the mean CT number for the contrast agent was significantly higher in 100 kVp images than in Sn 140 and in composite 120 kVp images (*P* < 0.001), and also significantly higher in composite 120 kVp images than in Sn 140 kVp images (*P* < 0.001). The mean CT number for oil was significantly lower in 100 kVp images than in Sn 140 kVp and in composite 120 kVp images (*P* < 0.001). The mean CT number for oil was also significantly lower in composite 120 kVp images than in Sn 140 kVp images (*P* < 0.001). The mean CT numbers for water differed among the images, but the trend did not reach clinical significance.

The mean CT values for the 3 materials were also compared among mononergetic images reconstructed at 40, 70, 100, 130, 160, and 190 keV (Table 1). Typical monoenergetic images of the phantoms are shown in Figure 3 (bottom rows). As the effective energy increased, the brightness of oil increased whereas the contrast agent decreased in brightness. Water appeared unchanged. The mean CT number for the contrast agent was the highest in the 40 keV images and decreased as the energy level increased (*P* < 0.001). The mean CT number for oil was the lowest in the 40 keV images and increased concomitantly with the increase in energy level (*P* < 0.001). The difference in mean CT number for oil between the 40 and 190 keV images was greater than 100 HU. The mean CT numbers for water differed among the monoenergetic images, but the trend did not reach clinical significance.

### 3.2. Clinical Study

#### 3.2.1. Patient Characteristics

The datasets of 17 patients (9 men and 8 women; mean age, 60.3 ± 10.2 years) were included. The mean body mass index (BMI) and heart rate were 22.3 ± 3.5 kg/m^2^ and 53.4 ± 6.1 bpm, respectively. The contrast volume per patient was 57.9 ± 8.1 mL. The mean coronary calcium score was 141.1 ± 274.1 Agatston score equivalents.

#### 3.2.2. Polyenergetic Imaging

**Figure 4:**
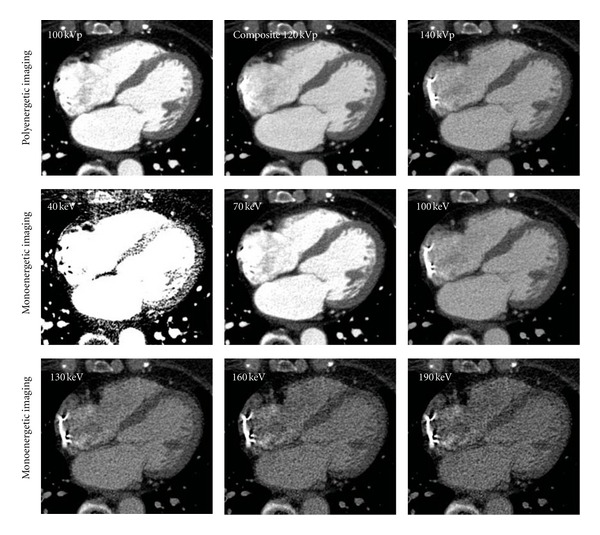
Clinical images; typical polyenergetic (top row) and monoenergetic (bottom rows) axial cardiac images. All images are displayed with a window width of 600 HU and window level of 200 HU. In the polyenergetic images, the cardiac cavity was most clearly visualized in the 100-kV image and darkened with the progressive increase in tube energy. In the monoenergetic images, the cardiac cavity and vertebrae appeared the brightest in the 40 keV image and darkened with the progressive increase in effective energy.

The mean CT numbers of several tissues were compared among the 100, Sn 140, and composite 120 kVp images (Table 2). Typical polyenergetic axial cardiac images are shown in Figure 4 (top row). The cardiac cavity was most clearly visualized in the 100 kVp image and darkened with the progressive increase in tube energy. The mean CT numbers for the left ventricular cavity, myocardium, and vertebrae were significantly higher in the 100 kVp images than in the Sn 140 and composite 120 kVp images (*P* < 0.001). The mean CT numbers for these tissues were also significantly higher in the composite 120 kVp images than in the Sn 140 kVp images (*P* < 0.001). In contrast, the mean CT number for pericardial fat was significantly lower in the 100 kVp images than in the Sn 140 kVp and composite 120 kVp images (*P* < 0.001) and was also significantly lower in the composite 120 kVp images than in the Sn 140 kVp images (*P* < 0.001).

#### 3.2.3. Monoenergetic Imaging

The mean CT numbers for several tissues were compared among the monoenergetic images reconstructed at 40, 70, 100, 130, 160, and 190 keV (Table 2). Typical monoenergetic axial cardiac images are shown in Figure 4 (bottom rows). The cardiac cavity and vertebrae appeared the brightest in the 40 keV images and darkened with the progressive increase in effective energy. The mean CT numbers for the left ventricular cavity, myocardium, and vertebrae were the highest in the 40 keV images (*P* < 0.001) and decreased as the energy level increased. In contrast, the mean CT number for pericardial fat was the lowest in the 40 keV images (*P* < 0.001) and increased with increasing energy levels. The difference in the mean CT number for pericardial fat between the 40 and 190 keV images was approximately 100 HU.

## 4. Discussion

This study represents the description of the influence of effective energy on CT number and the variance associated with different materials or tissues in cardiac monoenergetic images. The following are primary features common to both polyenergetic and monoenergetic images. Imaging of phantoms illustrated that the CT numbers for oil and the contrast agent significantly change depending on the energy level. As the energy level increased, the CT number increased in oil whereas it decreased in the contrast agent. Clinical imaging demonstrated that the CT numbers for several tissues change significantly depending on the energy level. The CT numbers for the left ventricular cavity, myocardium, and vertebrae decreased as the energy level increased. In contrast, the CT number for pericardial fat increased with increasing energy level.

The observed changes in CT number for the contrast agent associated with changes in the energy level were consistent with a previous report [3], with a phantom study that used rapid kV switching with a second-generation single-source CT scanner [6]. Phelps et al. reported changes in CT numbers for various body tissues (gray and white matter, fat, liver, pancreas, and muscle), fluids (water, cerebrospinal fluid, plasma, and red blood cells), and cerebral lesions (blood clots, edematous brain tissue, and brain tumors) [7], which are compatible with our results.

The changes in CT number for oil and for the pericardial fat in monoenergetic images are clinically relevant. In general, identification of adipose tissue has been primarily performed via measuring the absolute CT number and by assessing its location in polyenergetic images [7]. The results of this study suggest that identification of adipose tissue can be performed not only by the conventional methods but also by assessment of the change in CT number with respect to the effective energy in monoenergetic images.

The importance of adipose tissue evaluation is increasing in cardiology. Pericardial fat is a marker of adiposity and cardiovascular risk [8]. Subendocardial fat deposition, also called lipomatous metaplasia, is related to a history of myocardial infarction [9, 10]. Fat infiltration in the right ventricular free wall is an important finding for the diagnosis of arrhythmogenic right ventricular dysplasia/cardiomyopathy [9]. Moreover, the evaluation of lipids in atherosclerotic plaque is very important for the management of patients with coronary artery disease [11]. Relatively large amounts of adipose tissue can be sufficiently evaluated through measurement of the absolute CT number, but a small amount of lipid in a coronary atherosclerotic plaque is currently difficult to evaluate for the following 2 reasons: (1) the measured CT numbers for coronary plaque are affected by high intravascular attenuation as a result of high iodine concentrations [12] and (2) overlaps exist between the CT numbers of lipid-rich and fibrous plaques [13, 14].

Although monoenergetic imaging was not used, Tanami et al. obtained human coronary arteries at the time of autopsy and scanned them at different tube voltages (80, 100, 120, and 140 kVp) with single-source CT [14]. They evaluated the association between tube energy and the CT numbers for coronary plaques. As the tube energy increased, the CT number increased in lipid-rich plaques (confirmed by pathology), whereas the CT number decreased in calcified plaques. These results partially support the results of the current study and suggest that the assessment of changes in CT number associated with changes in the effective energy in monoenergetic images can be used to characterize coronary plaque. However, further investigations are required to confirm the utility of this approach in a clinical setting.

The results of this study must be evaluated in light of some limitations. First, the retrospective nature of this study was a potential limitation, and the evaluated data were from a small cohort at a single center. Second, we excluded patients with coronary artery stenoses considered severe enough for revascularization therapy and no prior percutaneous coronary intervention or coronary artery bypass grafting, for the accurate assessment of CT number change of the myocardium. Third, only contrast-enhanced monoenergetic images were evaluated. 

## 5. Conclusion

The influence of effective energy on CT number varies with the type of tissue in monoenergetic cardiac imaging. This suggests that monoenergetic cardiac imaging can be used to evaluate tissue characteristics not only by the measurement of the absolute CT number but also via assessment of the change in CT number associated with changes in the effective energy.

## Figures and Tables

**Table 1:** The CT number changes of three materials according to the energy level.

Polyenergetic images						
Tube voltage (kVp)	100		120		140	
Water (HU)	−3.1 ± 0.3		−2.2 ± 0.5		−1.8 ± 0.6	
Oil (HU)	−135.1 ± 0.4		−119.3 ± 0.2		−104.0 ± 0.5	
Contrast agent (HU)	682.0 ± 0.5		505.8 ± 0.3		329.0 ± 0.1	

Monoenergetic images						
Effective energy (keV)	40	70	100	130	160	190

Water (HU)	−7.0 ± 1.0	−2.8 ± 0.4	−1.7 ± 0.6	−1.3 ± 0.7	−1.1 ± 0.8	−1.0 ± 0.8
Oil (HU)	−228.1 ± 2.9	−127.0 ± 0.2	−101.0 ± 0.6	−91.9 ± 0.8	−87.9 ± 0.9	−85.9 ± 1.0
Contrast agent (HU)	1738.0 ± 1.7	590.5 ± 0.5	294.7 ± 0.2	191.2 ± 0.2	146.3 ± 0.2	123.7 ± 0.2

CT: computed tomography.

**Table 2:** The CT number changes of several tissues according to the energy level.

Polyenergetic images						
Tube voltage (kVp)	100		120		140	
Left ventricle (HU)	459.7 ± 84.5		358.7 ± 62.5		257.2 ± 41.2	
Myocardium (HU)	100.9 ± 20.4		86.4 ± 13.7		71.3 ± 9.2	
Pericardial fat (HU)	−96.1 ± 11.3		−86.2 ± 10.9		−76.8 ± 13.0	
Vertebrae (HU)	1034.0 ± 156.3		912.4 ± 140.7		790.1 ± 125.7	

Monoenergetic images						
Effective energy (keV)	40	70	100	130	160	190

Left ventricle (HU)	1218.0 ± 240.0	446.5 ± 78.7	247.6 ± 38.5	178.0 ± 25.7	147.7 ± 20.9	132.6 ± 18.9
Myocardium (HU)	211.4 ± 77.5	98.8 ± 19.1	69.7 ± 8.9	59.6 ± 9.7	55.1 ± 10.9	53.0 ± 11.6
Pericardial fat (HU)	−177.2 ± 43.8	−97.2 ± 10.8	−76.6 ± 12.1	−69.3 ± 14.2	−66.2 ± 15.4	−64.6 ± 15.9
Vertebrae (HU)	1949.0 ± 276.7	1019.0 ± 153.3	778.7 ± 124.2	694.8 ± 114.5	658.3 ± 110.5	640.0 ± 108.5

CT: computed tomography.
